# Supplementary material for: Relationships between Pulmonary Hypertension Risk, Clinical Profiles, and Outcomes in Dilated Cardiomyopathy
Source: J Clin Med. 2020 Jun 1;9(6):1660. doi: 10.3390/jcm9061660 (PMC7355437; doi:10.3390/jcm9061660)
Supplement: Supplementary file 1 [file jcm-09-01660-s001.pdf]

Table S1. Previous studies analyzing PH in DCM.

**Main author**	**Romeo et al.**	**Rihal et al.**	**Grzybowski et al.**	**Zhang et al.[3]**	**Hirashiki et al. (H-2014)**	**Hirashiki et al. (H-2016)**	**Li et al.**	**Mene-Afejuku et al.**	**Chen et al.**	**Bianco et al.**	**Own study**
Reference	[30]	[34]	[27]	[31]	[35]	[28]	[29]	[33]	[26]	[32]	-
No. of DCM patients	104	102	144	112	256	90	1119	351	35	81	502
Date(s) of study	1977–1987	1986–1990	1981–1991	2007–2009	2000–2011	2016[1]	2003–2011	2006–2016	2012–2014	2016–2017	2010–2020
Study Location	Italy	USA	Poland	China	Japan	Japan	China	USA[6]	China	Italy	Poland
PH identification	R	E	E	R	R	R	E	E	R	R	E
PH prevalence of PH (%), mean PASP (mmHg) or TRV (m/s)	25mmHg	2.9m/s	41mmHg	54%	14%	17%	18%[5]	41mmHg	63%,	73%, 50mmHg	2.6m/s
Mean age (years)	44	61	39	NA	52	52	51	62	48	64	54
NYHA III/IV (%)	49	34	73	81	10[3]	1.7[4]	73	NA	80	NA	49
Duration of symptoms (months)	35	NA	27	NA	NA	NA	24	NA	62	NA	39
Mean EF (%)	32	23	25	32	37	30	32	<40	29	26	26
Mean LVEDd (mm)	NA	69	75	NA	61	NA	68	60	70	NA	66
BB / ACEi, ARB or ARNI usage (%/%)	0/0	3/14	NA	NA	68/58	87/86	91/85	NA	NA	100/47	96/90
Comparison of PH and non-PH DCM patients (Yes/No)	N	N	N	Y	Y	Y	N	N	Y	Y	Y
Follow-up (years)	3.8	3	4.1	NO	4.3	NO	2.8	0.5	NO	NO	3.8
Death rate (%)	66	34	47		NA		24	28[7]			17

Designations: R – RHC, E – echocardiography, NO – no follow-up, NA – no information available. Notations: [1]date of publication (conducted retrospectively), [2]all patients with acute HF in last 3 months, [3]available only in Chinese (analysis based on an abstract in English abstract), [4]mean NYHA class, [5]PASP>40mmHg, [6]43% of the study population was African-American, [7]rehospitalization in 6 months. Abbreviations: DCM – dilated cardiomyopathy, PH – pulmonary hypertension, PASP – pulmonary artery systolic pressure, TRV – peak tricuspid regurgitation velocity, NYHA – New York Heart Association class, EF – ejection fraction, LVEDd – left ventricle end-diastolic, USA – the United States of America.

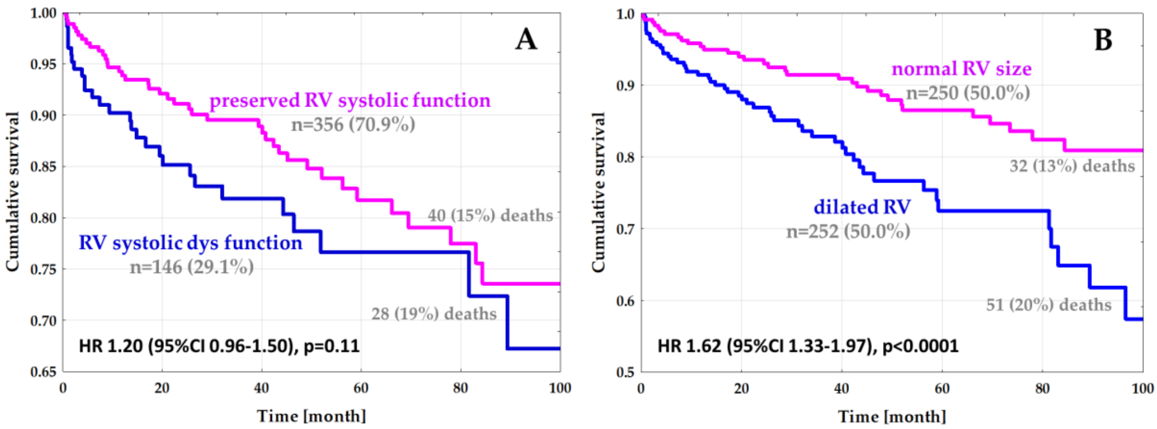

Figure S2. Kaplan–Meier estimates for all-cause mortality. Study population divided according to RV systolic dysfunction (**A**) and RV dilatation (**B**).

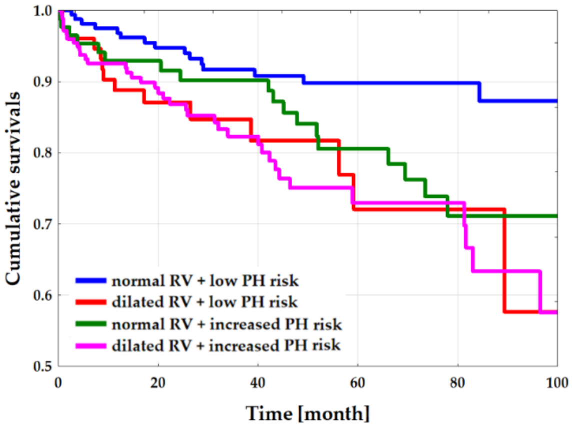

Figure S3. Kaplan–Meier estimates for all-cause mortality. Study population divided according to RV dilatation and low PH risk.
